# Supplementary material for: Risk of eczema, wheezing and respiratory tract infections in the first year of life: A systematic review of vitamin D concentrations during pregnancy and at birth
Source: PLoS One. 2020 Jun 15;15(6):e0233890. doi: 10.1371/journal.pone.0233890 (PMC7295196; doi:10.1371/journal.pone.0233890)
Supplement: S1 Table — (PDF) [file pone.0233890.s001.pdf]

Table S1: Search strategy worksheet for PubMed, MEDLINE, ProQuest, Scopus, CINAHL, the Cochrane Library and Academic Search Premier, March 2019

Concept 1		Concept 2		Concept 3		Concept 4
Vitamin D		Eczema		Respiratory Sound		Infection, Respiratory Tract
OR		OR		OR		OR
Vitamin D deficiency		Dyshidrotic eczema		Sound, Respiratory		Respiratory Tract Infection
OR		OR		OR		OR
Ergocalciferols		Atopic dermatitis		Breathing Sounds		Respiratory Infections
OR		OR		OR		OR
Cholecalciferol		Contact dermatitis		Breathing Sound		Infections, Respiratory
OR				OR		OR
Micronutrient			AND	Sound, Breathing		Infections, Respiratory Tract
OR				OR	AND	OR
Nutrition	AND			Sounds, Breathing		Upper Respiratory Tract Infections
OR				OR		OR
Dietary supplements				Lung Sounds		Infections, Upper Respiratory Tract
				OR		OR
				Lung Sound		Upper Respiratory Infections
				OR		OR
				Sound, Lung		Infections, Upper Respiratory
				OR		OR
				Sounds, Lung		Respiratory Infection, Upper
				OR		
				Stridor		
				OR		
				Stridors		
				OR		
				Rales		
				OR		
				Rale		
				OR		
				Crackles		
				OR		
				Crackle		
				OR		
				Pleural Rub		
				OR		
				Pleural Rubs		
				OR		
				Rub, Pleural		
				OR		
				Rhonchi		
				OR		
				Rhonchus		
				OR		
				Wheezing		
				OR		
				Wheezings		
