# Supplementary material for: Risk of eczema, wheezing and respiratory tract infections in the first year of life: A systematic review of vitamin D concentrations during pregnancy and at birth
Source: PLoS One. 2020 Jun 15;15(6):e0233890. doi: 10.1371/journal.pone.0233890 (PMC7295196; doi:10.1371/journal.pone.0233890)
Supplement: Attachment — Submitted filename: Response to Reviewers.docx [file pone.0233890.s003.docx]

Risk of eczema, wheezing and respiratory tract infections in the first year of life: a systematic review of vitamin D concentrations during pregnancy and at birth (Revised 2nd Edition)

“Author Response”

We would like to express our sincere gratitude to the esteemed reviewers for their useful comments. We have now revised the manuscript according to the reviewers’ suggestions, and sincerely hope that the manuscript is now suitable for publication in Plos One.

| **Editor Comment** | **Author Response** |
| --- | --- |
| Please ensure that your manuscript meets PLOS ONE's style requirements, including those for file naming. | Thank you for highlighting this. We had amended it accordingly. |
| *Please include in your methods section, the dates included in your search and the dates during which you performed your search. | The information that is requested already been updated in method section; Line 104-106  “The following databases were used to search for studies published until March 31, 2019; PubMed, MEDLINE, ProQuest, Scopus, CINAHL, the Cochrane Library and Academic Search Premier. This search was performed on May 1, 2019.” |
| *Please explain whether you assessed the heterogeneity of the manuscripts. | Thank you for pointing that out.  We did not use any scoring to assess heterogeneity. However, from the definition of outcomes evaluation, assessment for heterogeneity is by evaluating the 10 papers ( 8 studies reported the measurement of the outcomes were by parental reported and only 2 reported using established scoring tool. Hence because of that, we updated as limitation. |

| **Comments from Reviewer 1** | **Author Response** |
| --- | --- |
| The current study aims to systematically review the literature published until March 2019 on maternal vitamin D status in pregnancy and the development of eczema, wheezing and respiratory tract infections in the offspring within the first year of life. Although the literature search is comprehensive and includes a large number of databases, the rationale for the review in the context of previous studies is unclear, and the presentation of results is not reader friendly. The study also includes studies of cord blood vitamin D, which is not reflected in the title or objectives. | Thank you for pointing that out.  We appreciate the reviewer comments and we have revised some sentences, words, title and objectives of the review.  We have amended the title;  “Risk of eczema, wheezing and respiratory tract infections in the first year of life: a systematic review of vitamin D concentrations during pregnancy and at birth”  We also have updated the amendment and revision to PROSPERO with registration no. CRD42018093039. |
| Major comments: |  |
| 1. The introduction should reflect more strongly that this is a systematic review. | We acknowledged your input and the introduction had been revised accordingly to show this is a systematic review. |
| 2. Previous reviews and meta-analysis on the current topic need to be summarized and referenced before they appear in the Discussion (refs 26, 43, 44), and there should be a clear description of what the current study contributes. | Thank you for pointing that out.  The information have been added accordingly in the introductory section; Line 71-75  “To this day, it is still unclear to what extent the vitamin D levels during pregnancy or early postnatal (e.g, cord blood) affects the development of allergic diseases and RTIs in infants. Prior reviews have included a wide range of age while assessing allergic diseases and RTI outcomes (e.g 0 to 5 years old), and employed different methods in vitamin D measurement; serum 25(OH)D and dietary intake (4, 23, 26). |
| 3. The focus on RTI in the Asia-Pacific region creates some expectations that this review will add evidence from non-western populations, but this does not seem to be the case. | Thank you for highlighting this. It would be a good input if studies had been conducted in Asia Pacific region. We did not found any. A study conducted in Japan had to be excluded as had met our exclusion criteria. |
| 4. It is unclear why the atopic march is brought up, as the current study is limited to infants < =12 months. | Thank you for pointing that out.  We take note it and we had removed it. |
| 5. Greater emphasize could be placed on the potential role of maternal vitamin D status in the etiologies of eczema, wheezing and RTI in the offspring. | Thank you for highlighting this. Noted that vitamin D has potential role in bone’s health, other musculoskeletal health such as rickets, cardiovascular disease, diabetes and certain cancers. However it is not strong enough to show the association of high vitamin levels and the eczema status. Thus we could not put a greater emphasize literature on this issue.  We have gone through and make necessary changes. |
| 6. Also, the introductory description of the occurrence and time trends in eczema, wheezing and RTI in infants should be more precise with an appropriate choice of references, e.g. references 1 and 2 seem a bit arbitrary (USA) or old (WHO) for documenting the current situation. Judging from the titles, references 3-5 to do not appear to be primary sources of data showing an increase in the rates of over time. General statements such as “…54.7% have been diagnosed with RTIs in early life” is supported by a study from a regional hospital in Cameroon, which is insufficient. | Thank you for highlighting this.  We had removed that points and we had amended it. |
| 7. The reference list is incomplete with several journal names missing, so revision is needed. | Thank you for pointing that out.  We had revised and had corrected it. |
| 8. Search strategy and PICOS criteria (Table 1): the objective of the study is to review literature on maternal vitamin D status, but 4 of 10 included studies have assessed vitamin D status in cord blood. The title/scope/Table 1 PICOS statement and study selection criteria could be changed to reflect the inclusion of studies of vitamin D status measured at birth, or these 4 studies should be excluded from the paper. The PICOS statement for “outcome” should include children and age group. | Thank you for the reviewer comment and suggestion.  We had revised it and had made necessary changes.  We have amendment the title and had been changed accordingly.  The title/scope/Table 1 PICOS statement and study selection criteria had been updated and had amended in PROSPERO. |
| 9. Search terms (Table S1): It seems that the literature search could have been more targeted by including terms for pregnancy, infants or human studies (ref Table S1 PICO). The very broad search may explain why so few studies (10 of 2678 records) were included in the end. | Thank you for the suggestion. We used a larger search term to ensure we did not miss the evidence by specifically. Having specific search term, we afraid it may reduce paper that will be selected. |
| 10. It is unclear if search terms were MeSH and/or text words, and why vitamin D deficiency, but not excess (which has also been associated with the outcomes of interest) was included, or chemical forms of vitamin D. | Thank you for pointing that out. We used MESH term and this had already explicitly in line 109-111. |
| 11. Data extraction (line 131): data on the adjustment for maternal confounders other than parental history should also be extracted and considered when interpreting results and inconsistencies, e.g. were results adjusted for other nutrients of potential importance (e.g. fatty acids or folate) as referenced papers seem to indicate. | Thank you for highlighting this. We had added in the table result accordingly. |
| 12. Results/study characteristics: it would be helpful if the summary included number of studies vs publications for each outcome, and how these outcomes were assessed (e.g. maternal report or clinical diagnosis) before describing ethnicity of the study populations. | Thank you for highlighting this.  The information that is required already been updated.  “Methods of outcome assessment were diverse. Most studies relied on parental reports (n=8 studies) while some used standardized tools (n=2 studies). The two tools used to determine eczema were the ‘SCORing Atopic Dermatitis’ (SCORAD) index (7) and the modified version of the UK Working Party’s diagnostic criteria (35). |
| 13. Results in text: reference category needs be mentioned when reporting relative-risk estimates. | Thank you for highlighting this.  The information that is required already been updated. |
| 14. Results: Table 2 does not seem space efficient, or reader friendly. Maybe better if split into 2 tables (separate for study characteristics and results where similar outcomes are grouped together) | Thank you for highlighting this.  We appreciate the reviewer suggestion and we have simplified the table accordingly. |
| 15. Results/vitamin D status (line 180-182): a mean value of 14 nmol/L seems unlikely, please verify. | Thank you for pointing that out.  We take note it and had revised and made necessary changes. Line 186-187.  “The average concentration of serum 25(OH)D during pregnancy ranged 50.0 to 73.6 nmol/L and early postnatal (in cord blood) ranged from 44.0 to 58.4 nmol/L.” |
| Minor comments: |  |
| 1. Results: information regarding exclusion criteria in first paragraph is better suited under Methods. | Thank you for the reviewer comment.  The information has been removed and changes has been made accordingly in Method section. |
| 2. Results: Some parts of the Table 2 is confusing and may have errors. It seems that LRTI at 15 months is included (Camargo et al 2010) although the study selection criteria is <= 12 months. | Thank you for pointing that out.  Thank you for the reviewer comment.  The information had been removed and changes has been made accordingly. |
| 3. Under summary statistics for exposure, what is meant by “(Median 25(OH)D cord blood (IQR) nmol/L = n; 922”? | Thank you for pointing that out.  The information had been added. |
| 4. Abstract: unclear what level of maternal vitamin D that seems to be protective (line 36-37). Also not possible to conclude that evidence is inconsistent due to low number of studies. | Thank you for pointing that out.  We had revised and had made necessary changes accordingly. Line 39-42  “Studies on the associations between vitamin D concentrations during pregnancy and at birth and the mentioned outcomes among infants were found limited. Nevertheless, there is conflicting evidence showing the risk of eczema, wheezing and RTIs with vitamin D concentration in early life.” |
| 5. Line 189-190: unclear definitions | Thank you for pointing that out.  We have gone through and make necessary changes. Line 193 – 196.  “In determining the associations between exposure (vitamin D concentrations during pregnancy or early postnatal) and outcomes (allergic eczema, wheezing or RTIs), some studies used quartiles and quintile while some employed cut-off values with different presentation of vitamin D concentration categorizations, such as deficient (25.0-40.0, < 50.0 nmol/L),…” |
| 6. Line 184: incomplete sentence | Thank you for pointing that out.  The information had been added. Line 190-191.  “Three studies reported mean and standard deviation for the serum 25(OH)D results…” |

| **Comments from Reviewer 2** | **Author Response** |
| --- | --- |
| Overall this is an excellent piece of work and that helps to answer an important question. I think the methodology is in keeping with best practice, and the manuscript is largely well written with appropriate conclusions drawn from the results.  The main reason for "major revision" is that I feel there should be assessment and reporting of how outcomes were defined/measured in the methods and results section. As the authors state in the discussion, this can significantly impact the results and as such should be discussed. If that was adjusted, in addition to some other minor points below, then I would think the manuscript would be ready for publication. | Thank you for pointing that out.  We appreciate the reviewer suggestion and we have corrected and make necessary changes. The outcomes were defined as in Line 162 – 165. |
| 1. Abstract   - Clear and well written | Thank you for the comment. |
| 2. Introduction:  - The paragraph that begins Page 3, Line 58 is confusing. The paragraph starts with a description of the “atopic march” concept, and then says “similar patterns were observed in RTI cases”. However RTI cases in early life do not lead to more severe persistent RTI cases in later life, so I do not think there is a similar pattern to atopic march. I think what the authors are trying to say is that atopic disease and RTI are both common in early life, and important causes of morbidity as atopic disease can “march” on to more severe, persistent atopic disease and RTI is a leading cause of hospital admission. If so, I agree with that, but I think the paragraph needs reworking. This is a minor point, but the final paragraph of the introduction would have more of an impact of it consisted of the 2 sentences. The one the starts “to this day …” (page 4, line 82) and “this systematic review aimed …” (page 4 line 85). - The points re importance of measuring serum vitamin D rather than dietary is important and valid, but could be worked into the previous paragraph. | Thank you for pointing that out.  We have gone through and make necessary changes.  We have removed the information of “atopic march” paragraph. This has been amended and comment were incorporated in introduction section (Line 58-62).  Thank you for highlighting this. We had revised and had amended accordingly. Line 76- 79.  “Clinically, serum 25hydroxyvitamin D (25(OH)D) is used as a marker of vitamin D level because it represents the cumulative effect of dietary intake of vitamin D and sunlight exposure (27). Thus, dietary intake alone might not be a good indicator of overall vitamin D levels as ultraviolet B-rays induces skin formation of vitamin D.” |
| 3. Methods  - well done - clear question, followed best practice, registered prospectively - Please reference the table with the search terms in the data sources paragraph so that readers know it is available. - assessment of definition of each outcome is important as this is a potential source of heterogeneity (i.e was eczema /wheezing/RTI parent report, doctor diagnosed, or assessed by the trial team) | Thank you for the comment.  We take note it and had referenced the table with the search terms as in line 111.  The description on how outcomes were assessed by the studies had been updated as in line 162– 165. |
| 4. Results  - Please reference the PRISMA diagram at the start of the results section, so that readers know that is available for them to look at. Same for PRISMA checklist. - Need to incorporate comment on how each outcome was assessed. See feedback above | Thank you for pointing that out.  We take note it and had referenced the PRISMA diagram at the start of the results section as stated in line 155 – Figure 1. Figure 1 is the PRISMA diagram. |
| 5. Discussion  - minor point; page 22, line 261 “- when put together-“ would be better to have “,” rather than “-“ (i.e , when put together,) | Thank you for pointing that out and the suggestion.  We have corrected it. Line 294. |
| - page 23, line 280; It would be good to briefly outline what the results of prior systematic reviews are, that this review contradicts | Thank you for pointing that out.  We have gone through and make necessary changes. Line 308-310.  “Previous reviews indicated a preventive role of vitamin D in early life against wheezing and RTIs; inverse associations were found between vitamin D levels in early life and wheezing and/or RTIs among infants (23, 26).” |
| - page 23, line 283; matured should be mature | Thank you for pointing that out and the suggestion. We have corrected it. |
| - page 23, line 284; there is a full stop missing before “Second” | Thank you for pointing that out and the suggestion. We have corrected it. |
| - page 23, line 285; this is an important point (method of outcome assessment) but should not be mentioned for the first time here. It should be incorporated into both methods and results. | Thank you for pointing that out.  We have gone through and make necessary changes. We added information of method of the outcome assessment in Result/study characteristic section. Line 163 – 171. |
| 6. Conclusion  - Page 24; Line 314; Include examples of what more appropriate and comparable methodologies would include (i.e multiple measurements of vitamin D, with statistical analysis involving … and outcomes assessed …) | Thank you for pointing that out.  We have gone through and added a few points. Line 354-359.  “These include employing randomized control trial or cohort study design with a larger sample size, and measuring the outcomes based on medical diagnosis or established clinical tools to ensure its’ accuracy. In addition, the study subjects should comprise a more specific and targeted age group….” |

| **Comments from Reviewer 3** | **Author Response** |
| --- | --- |
| This is a well written perspective that discusses the evidence for maternal vitamin D levels controlling the incidence of eczema, wheezing and respiratory tract infections in infants before one year of life. The maternal levels were measured at times ranging from during the first trimester to ‘at birth’ with measures in the cord blood. Vitamin D levels from a single sample were sometimes published as absolute amounts but in other studies only defined into categories. | Noted with thanks. |
| Another limitation was that some outcomes were based on parental reports and were not necessarily nurse or doctor defined. | I agree with your comment and this is highlighted in our limitation section. |
| Ten studies were scrutinised in full, all of which included primarily western/Caucasian populations. Measures of maternal vitamin D were considered as a possible determinant of infant eczema, wheezing or respiratory tract infections; the discussion for this study did not consider vitamin D levels of the infant per se as a possible contributor to allergic outcomes. Perhaps vitamin D deficiency during the first year of life is as important as the level before birth. The review is informative and suggests that no robust association between maternal vitamin D and infant allergy exists. If an effect exists, it is minor and can be detected only inconsistently in studies. It will be interesting if the authors perform a similar analysis in the Malaysian population; such a study would be very informative due to the latitude of Malaysia and the Malaysians recruited to the study will have obtained their vitamin D levels from significant sun exposure. | Your constructive suggestions is noted and the study among infant in Malaysia will be conducted in near future. |
| Many of the other studies reported included populations living at higher latitudes. As inferred in line 89, there is a growing awareness that vitamin D levels are a biomarker of recent sun exposure rather than being the active molecule in the condition under study. Any association of vitamin D levels with a condition cannot infer that vitamin D is the causal molecule. Instead, other sun-induced molecules may be responsible for the benefit found. In a revised manuscript, this argument should be included in the Discussion. | Thank you for the suggestions. I agreed that potentially there are other molecule that is activated by sun that may confer benefits. However the literature on this is scarce. Nevertheless, despite some countries at the equator and have sun exposure most of time, the population/ the subject can also be at risk of vitamin D insufficiency due to the clothing style as reported by recent study, Quah et al. (2018). |
| - Line 151, remove with. | Thank you for pointing that out. We have removed “with”. |
| - Line 184, something is missing, Three what? | Thank you for pointing that out. We have added the missing sentence. |
| - Line 280, references are required for the prior systematic reviews. | Thank you for pointing that out.  We take note it, and had added the references. |
