# Supplementary material for: Risk of eczema, wheezing and respiratory tract infections in the first year of life: A systematic review of vitamin D concentrations during pregnancy and at birth
Source: PLoS One. 2020 Jun 15;15(6):e0233890. doi: 10.1371/journal.pone.0233890 (PMC7295196; doi:10.1371/journal.pone.0233890)
Supplement: Attachment — Submitted filename: Response to reviewers.docx [file pone.0233890.s004.docx]

Risk of eczema, wheezing and respiratory tract infections in the first year of life: a systematic review of vitamin D concentrations during pregnancy and at birth (Revised 3rd Edition)

“Author Response”

We would like to express our sincere gratitude to the esteemed reviewers for their useful comments. We have now revised the manuscript according to the reviewers’ suggestions, and sincerely hope that the manuscript is now suitable for publication in Plos One. Below are the author response to the reviewer comments.

Reviewer #1: Comments to paper after first revision:

| **Comments from Reviewer 1** | **Author Response** |
| --- | --- |
| Although the methodology for the literature search seems solid, the revised manuscript and presentation of results seem to suffer from typos, errors and inconsistencies and leaves an untidy impression.  Also, it still remains unclear what the current study adds compared with the previous reviews and meta-analyses referenced (refs 4, 23, 26); e.g. does the current study include more studies on wheeze in infants than the meta-analysis from 2018 (ref 4) or do you summarize the evidence differently? | Thank you for highlighting this. We had amended it accordingly.  The information that is requested already been updated in method section; Line 80-98 |
| The authors argue that previous reviews have included vitamin D intake, but this is not a problem as long as vitamin D intake and vitamin D status are presented as separate analyses, which is usually the case –without knowing these studies in detail. Regarding the age restriction of all outcomes (RTI, eczema, wheeze) to infants, the rationale for doing this should be made more clear, especially because the authors conclude that the literature is limited and the outcomes of eczema and wheeze also occur in children after the first year of life. Some arguments now mentioned in the Discussion, could be moved to the Introduction. However, for vitamin D there is still a chance that prenatal exposure may play a role, due to vitamin D supplement recommendations to infants in some countries, and the introduction of complementary feeding which may be enriched with vitamin D. | The information that is requested already been updated in method section; Line 80-84; 92-95. This is further elaborated to highlight the importance of current literature review. |
| ***Major comments*** |  |
| The study rationale could be refined some more. | The information that is requested already been updated in method section; Line 80-84; 92-98. |
| The manuscript needs proof reading from the very beginning, a few examples include the Abstract (line 29 – duplicate words, missing commas; line 36 missing “in” or “during”) and Introduction (line 50, grammar). Clarity is affected in some places, e.g. in the Introduction (line 57) – it is unclear if “allergy eczema” should read “allergy and eczema” or “allergic eczema”. There are now 2 tables labelled “Table 3”, including the second page of Table 2, which should be corrected. | Thank you for highlighting this. The manuscript was proofread. We had amended and corrected it accordingly. |
| The paper is currently missing some information on outcome definitions as part of the inclusion/exclusion criteria. What types of eczema, wheeze, and RTI were included in the analysis and how were the outcomes assessed?  Results on eczema (Table2/Table 3) seem to be presented for both eczema and atopic eczema, and RTI (Table 5) seems to include several types, including LRTI (pneumonia/bronchiolitis), URTI, recurrent RTI, as well as symptoms of cough. Now the information is briefly mentioned in the Discussion (line 286, 287).  Unclear why ”Pregnancy outcomes” are mentioned in Table 2/3 (ref 38). | The information that is requested already been updated in method section; Line 128-131; 148-151  Thank you for highlighting this. We had removed it. |
| Tables have improved, but the information seems to be provided in an inconsistent manner. If the information was not available, it should be explained somewhere. The conclusion should preferably reflect the conclusions of the authors of the review (not the conclusions found in the papers), and be reported in a similar manner for all papers to the extent possible.  Taking Table 3 as an example:  Gale et al: no sample size provided, sig results for Q4 not marked in bold (but some results marked in bold in Table 4)  Weisse et al: no sample size provided, unclear if adj ORs refer to highest vs. lowest category  Gazibara et al. unclear if “n” refers to no. of cases or study size  Baiz et al. sample size provided twice (n=239), unclear if the reported OR is per nmol/L of vitamin D or if exposure categories are missing | Thank you for highlighting this. We had amended it accordingly.  Thank you for highlighting this. We had corrected and bold it.  The information already been updated.  Thank you for highlighting this. We had amended it. We also added a footnotes. N=study size; n=number of cases.  We had removed it and the information been updated in the table. |
| Summary of results: it is hard to follow at times. The authors could consider summarizing the number of studies reporting no/positive/negative associations for each outcome. | Thank you for the suggestions. We had amended it accordingly. |
| Discussion/Vit D & RTI: although sex of the child may affect the risk of RTI, sex is not expected to be associated with maternal vit D status or vit D status at birth, and therefore not a confounder by the usual definition. Thus, lack of adjustment for sex should not be considered a weakness of studies. | Thank you for highlighting this. We had removed and amended it accordingly. Line 315-321. |
| ***Minor comments*** |  |
| Reference no. 9 to document prevalence (lines 52, 56), is based on mathematical modelling and not original data, which should be stated explicitly | Thank you for highlighting this. We had amended it accordingly. Line 56-59. |
| Table 4: could go in the supplement with Table S3 |  |
| Tables 3-5 titles: Suggest changing for better clarity from “Studies included in the analysis between 25 (OH)D during pregnancy and cord blood and the development of … in the first year (12 months) of life” to “Studies included in the analysis of 25 (OH)D during pregnancy or cord blood with the development of … in the first year (12 months) of life”. | Thank you for the suggestion. We had amended it accordingly. |
| Discussion/Vitamin D status: unclear what is meant by “evidence has shown that vitamin D concentrations are a biomarker of sun exposure” because you also write that vitamin D concentrations reflect the “cumulative effect of vitamin D dietary intake and sun exposure”. | Thank you for highlighting this. We had removed the sentence and the sentences were rephrased accordingly. Line 273-282. |

Reviewer #2:

| **Comments from Reviewer 2** | **Author Response** |
| --- | --- |
| Thanks you for the revisions. I think you addressed most of my concerns. There are a few minor points;   1. Page 10 - heading of the table is "Table 3 Characteristics of 10 studies included in the analysis between 25 (OH)D during pregnancy and cord blood and the development of eczema, wheezing and RTIs in the first year (12 months) of life (continue)" - should this be table 2 (not table 3)? | Thank you for highlighting this. We had renumbered it accordingly. |
| 2. I think it would be good to include a column in Table 2 on how the each outcome was assessed, even if it is just health professional diagnosed or parent reported. | Thank you for the suggestion. We had added it accordingly in Table 2. |
| 3. I think the results section, under the headings Vitamin D and Eczema in the first year (12 months) of life, Vitamin D and wheezing in the first year (12 months) of life, and Vitamin D and respiratory infections in the first year (12 months) of life it would be good to comment on whether the method of outcome assessment could explain the conflicting results found. | Thank you for the suggestion. We had added the information accordingly. Line 220-222; 226-227. |

Reviewer #3:

| **Comments from Reviewer 3** | **Author Response** |
| --- | --- |
| This is an improved manuscript but the English spelling and phrases are very poor. Much of the manuscript requires rewriting. Many of the corrections required are listed below but perhaps an English writer should be consulted.  Line 36: During should be removed.  Line 50: continued to increase.  Introduction: 3rd sentence is repetitive of the 2nd sentence.  Line 56: years 2014 to 2015.  Line 57: ranged from xx  Line 62: The majority.  Line 64: second para, reword the sentence.  Line 66: ‘low’ vitamin D status  Line 68: remove ‘the’  Line 72: Cord blood does not represent early postnatal. In many places (for example line 107), cord blood is clearly stated as at birth, rather than early postnatal.  Line 81: remove course  Line 87: remove pool  Line 88: Low vitamin D levels are the risk factor  Line 129: ‘the’  Line 187: ranged ‘from’  Line 188: the meaning of ‘timing of exposure assessment’ is not clear  Line 210: Meaning of late of pregnancy?  Line 263: remove ‘There are’  Line 268: It is associations between X and Y  Lines 278-280: rewrite as meaning not clear  Line 282: populations  Lines 287-288: The literature is not scarce for the properties of other molecules induced by UV radiation. See Hart et al, Nat Rev Immunol 2011, and Hart et al, Annu Rev Pathol 2019.  Lines 288-290: not good English, please rewrite. | Thank you for highlighting this. We had corrected amended it accordingly.  Thank you for highlighting this. Noted with thank you. We had amended it. |
