# Supplementary material for: Risk of eczema, wheezing and respiratory tract infections in the first year of life: A systematic review of vitamin D concentrations during pregnancy and at birth
Source: PLoS One. 2020 Jun 15;15(6):e0233890. doi: 10.1371/journal.pone.0233890 (PMC7295196; doi:10.1371/journal.pone.0233890)
Supplement: Attachment — Submitted filename: Response to reviewers.docx [file pone.0233890.s005.docx]

Risk of eczema, wheezing and respiratory tract infections in the first year of life: a systematic review of vitamin D concentrations during pregnancy and at birth (Revised 3^rd^ Edition)

“Author Response”

We would like to express our sincere gratitude to the esteemed reviewers for their useful comments. We have now revised the manuscript according to the reviewers’ suggestions, and sincerely hope that the manuscript is now suitable for publication in Plos One. Below are the author response to the reviewer comments.

Reviewer #1: Comments to paper after first revision:

| **Comments from Reviewer 1** | **Author Response** |
| --- | --- |
| The authors have done a thorough revision. The study rationale is much now clearer and the manuscript reads a lot better. Most of my comments have been addressed and tables have improved considerably. A few minor points remain regarding the reporting of the results.  Table 2: no apparent order of studies. Could be sorted by publication year. | Thank you for the comment. We had amended it accordingly.  Thank you for the suggestion. We had sorted the information in Table 2 by the recent year of publication accordingly. |
| The marking of significant ORs in bold does still not seem entirely consistent, e.g. in Table 4 significant ORs are unmarked for the studies by De Jongh (OR for Q2) and Baiz. In Table 5 the same applies to the studies by De Jongh (Q3) and by Camargo. | We appreciate the comments and we had bold the information and corrected it accordingly. |
| It remains unclear for some studies what unit/increase the ORs are reported for, e.g. Table 4 for the studies by Jones and by Baiz. In Table 5 the OR is reported for a 10 nmol/L increase in the study by Palmer, which is the preferable reporting style. | We are sorry for the missing information. The information that is requested already been updated in the Table 4. |
| The reporting of the results from the study by Weisse (Table 3) are slightly confusing as the quartiles include case numbers and not ORs as for the other studies, and it remains unclear if the single OR estimate refers to any of the categories (low vs high or opposite) or a continuous effect.  I suggest changing “positive association” for the study by De Jongh to “Low maternal serum 25(OH)D levels decreased the risk of… ” which is more consistent with the reporting of other studies, and easier to understand. | Thank you for highlighting this. We did re-checking the reporting result of Weisse et al. (2013) study. The study reported they performed multivariate logistic regression and presented case numbers in each quartiles instead of ORs as for the other studies. As regards to eczema outcome definition, the eczema was recorded as parental report of symptoms and also parental report of a doctor diagnosed. Therefore, there are two results of eczema were presented.  Thank you for the suggestion. We had changed the sentence as suggested accordingly. |
| ***Other comments*** |  |
| Line 87-88: Reference has been inserted as author/year, but should be a number to be consistent with style.  Title of Tables 4 and 5: suggest replacing "and cordblood" with "or cordblood" as in title of Table 3  The unit of vitamin D concentration (nmol/L) seems to be converted from the original units in the paper in one or more studies (e.g. Weisse 2013 reported in ng/mL) – should be mentioned under Methods if the reported units differ from the original study. | Thank you for highlighting this. We had amended it accordingly. Line 88.  Thank you for the suggestion. We had changed the phrase as suggested in title of Table 4 and 5 accordingly.  Thank you for highlighting this. After further discussion among the authors, we had changed the converted ones to the original units in the papers. However, for clarity purposes for comparison (the ng/ml change to nmol/L), we put a footnote, 1 unit ng/ml equivalent to 2.496 nmol/L. The information already been updated in footnote each table accordingly. |

Reviewer #3:

| **Comments from Reviewer 3** | **Author Response** |
| --- | --- |
| It is difficult to grasp the aims and originality of this analysis. Lines 133-138 detail the aims of the analysis. However, after reading multiple times, I cannot distinguish the difference between aims 2 and 3. No effect is measured, all the studies included are associations.  The authors concluded that there were no associations between 25(OH)D levels during pregnancy and at birth, and 3 respiratory based outcomes in infants up to one year of age. The relevant discussion that address why this may be so has been removed from the document. 25(OH)D is a biomarker of being in the sun but it is not necessarily the active molecule for the outcomes investigated. This important point has been removed. Reasons for a lack of association requires discussion.  Lines 58, 274, 277, Asian countries not Asia countries  Line 58, remove while.  Line 55, prevalence is singular and requires a singular not plural verb. | We are sorry for the confusion of the information. To avoid the misunderstanding, we had removed the goal of no.3 as our review summarized and elaborated the findings in a narrative manner.  Thank you for highlighting this. The information that is requested already been updated accordingly. Line 288-291.  Thank you for highlighting this. We had amended it accordingly. Line 59, 277, 286, 289  Thank you for highlighting this. We had removed ‘while’ at line 58.  Thank you for highlighting this. We had amended ‘were most common…’ to ‘was most common..’. Line 55. |
